# Use of dislodgeable foliar residue studies for the evaluation of isomerization potential of active substances for non-dietary risk assessment

**DOI:** 10.1371/journal.pone.0312688

**Published:** 2024-11-01

**Authors:** Silke Wagner, Christian J. Kuster

**Affiliations:** Crop Science Division, Bayer AG, Monheim, Germany; University of Lucknow, INDIA

## Abstract

When assessing the non-dietary risks for operators, workers, bystanders and residents to active substances in plant protection products (PPPs) that contain stereoisomers, the EFSA guidance on stereoisomers recommends the application of uncertainty factors when the initial ratio of stereoisomers undergoes a significant stereoisomeric excess change of more than 10%. This precautionary approach may be over-conservative in cases where the change in isomers is due to differences in their degradation rates rather than conversion of one isomer to a potentially more toxic isomer. Therefore, the impact of isomeric composition in non-dietary risk assessments of PPPs was evaluated, with particular emphasis on dislodgeable foliar residues (DFR) data and potential enantioselective degradation processes. Hypothetical outcomes are discussed as well as an evaluation of data for 5 compounds from a total of 35 DFR studies conducted under GLP, aimed at deriving DFR values for higher tier assessments. The findings indicate that possible isomerization of the active substance on leaves can be detected in DFR studies, which is essential for assessing risks to workers and residents. The results of this evaluation highlight the potential for over-conservatism of the current EFSA method for calculating uncertainty factors in non-dietary risk assessments. The EFSA method’s reliance on worst-case assumptions, coupled with the lack of a formal statistical basis, can lead to overestimation of exposure, as illustrated in our theoretical considerations and supported by empirical data from DFR studies. To address this, we propose an alternative approach to evaluating risk considering both degradation and interconversion rates.

## Introduction

Pesticides or Plant Protection Products (PPPs), play a vital role in agriculture as they control pests that reduce crop yields [1]. Nevertheless, as for all chemicals, the potential direct and indirect effects of PPP use on human health and the environment necessitate comprehensive risk assessments [2–4]. Hence, binding guidelines are in place to govern the proper conduct of these risk assessments, ensuring the safe use of PPPs for both humans and the environment when used according to the label. With regard to non-dietary human exposure assessment in Europe, the European Food Safety Authority (EFSA) has developed a guidance document [5]. This document aims to estimate and mitigate the impact on operators handling the PPPs, workers re-entering treated crops, and bystanders or residents, whose exposure is incidental and unrelated to the PPP application. Each risk assessment considers the exposure to a PPP at the time of or after application by comparing it to the appropriate toxicology endpoint of the active substance (AS), thus ensuring the responsible and safe use of the PPP.

ASs used in PPPs may exist as isomers, such as enantiomers, which share the same molecular formula but are mirror images of each other and not superimposable. Although enantiomers have identical physicochemical properties, their biological activity and toxicity may differ due to interactions with enzymes or other naturally occurring asymmetric compounds. Therefore, one enantiomer may function effectively as a PPP, while its mirror image may be inactive as a PPP or present unintended toxicity, or vice versa. Additionally, enantiomers may exhibit different degradation rates, causing one enantiomer to be more persistent than the other [6]. Such enantioselective behaviours impact the risk assessment of chiral PPPs, necessitating an understanding of the individual enantiomers’ fate and behaviour in the environment, and their corresponding effects on humans and other non-target organisms.

The existing EFSA guidance for the consideration of isomers in risk assessments [7] states that stereoisomers should be regarded as separate chemical constituents in the risk assessment process, to ensure that all potential risks to humans and other non-target species are thoroughly addressed. However, the guidance does not explicitly address non-dietary exposure scenarios, particularly those involving dislodgeable foliar residues (DFR).

Hazard data for individual isomers and/or stereospecific exposure data are often unavailable; therefore, the EFSA guidance suggests methodologies for addressing different scenarios involving compounds with one or more stereogenic elements. Regarding non-dietary exposure, the primary groups impacted by changes in stereoisomeric composition after applying the product include workers and residents, with main exposure routes being through contact with treated foliage due to re-entry activities, vapor and surface deposits. Because isomerization–if it occurs–requires time, operators and bystanders are typically not the focus as they are exposed to either the concentrated products (with known isomeric content and tested in toxicological studies) or their dilutions, respectively. The composition of the AS at the point of application can be presumed to maintain the authorized ratio of stereoisomers, e.g., since the stability of the formulated PPP must be demonstrated by the applicant [8] and Dislodgeable Foliar Residue (DFR) data presented in this paper underline this approach. Nevertheless, for workers and residents exposed to residues on e.g., leaf and other crop surfaces, after the application of PPPs a potential for preferential degradation or shift in the stereoisomeric ratio cannot be completely disregarded.

The EFSA guidance suggests that when the initial ratio of stereoisomers undergoes a change of the stereoisomeric excess of more than 10% compared to the original composition, an additional uncertainty factor should be added in the risk assessment. This factor addresses the potential difference between the toxicologically tested isomeric mixture and the actual residue someone might be exposed to. If ASs and PPPs do not contain a mixture of stereoisomers (either the AS itself has no stereogenic element or is a single isomer) but generate transformation products with possible stereoisomers, the concept then needs to be applied on the metabolites, transformation products, respectively.

For workers, the EFSA guidance on non-dietary exposure [5] utilizes the DFR data to estimate dermal exposure for both workers and residents during the re-entry scenario. For turf surface deposits, the turf transferable residue (TTR) is used, which parallels a DFR for residues of PPPs deposited on lawns. Studies on residues in plants and environmental fate studies are not referenced in the OPEX guidance [5] as relevant scenarios for risk assessment for workers or residents. To assess potential changes in the stereoisomeric excess the guidance on stereoisomers notably does not explicitly refer to DFR trials but highlights instead residue data for crop and environmental matrices. However, DFR trials are capable of effectively detecting potential shifts in the stereoisomeric composition of a PPP. This is because stereospecific measurements are performed using validated methods, and the results are adjusted based on field trial recoveries. These trials are the basis to determine risk in exposure scenarios for workers and residents under real field conditions.

In this study, we examine the significance of isomeric composition in non-dietary risk assessments of PPPs. We start with hypothetical considerations, emphasizing scenarios where an increased number of isomers could affect risk assessments. Then, we compare these hypothetical outcomes with measured DFR data, focusing on potential enantioselective degradation processes. Additionally, in recognition of the unique characteristics of DFR studies and their relevance for assessing risks to workers and residents, we propose a tailored approach that considers both degradation and interconversion rates of stereoisomers to assess the relevant risk for individuals coming into contact with isomerized PPPs on the leaf surface. This approach also considers the high variability of results typically observed under real field conditions, where amounts of residues could exceed the 10% threshold of isomeric change without being significant and/or relevant. Lastly, in this evaluation, we model the decrease of residues on the leaf surface, aiming to determine the conditions under which a potential risk caused by isomerization could occur.

## Materials and methods

The analysed dataset is composed of 35 DFR studies conducted by Bayer between 2011 and 2021. All studies were conducted according to Good Laboratory Practice (GLP) which ensures transparent and verifiable results. These studies were designed to refine product-specific risk assessments. The research was performed in both the northern and southern residue zones in Europe (as defined by residue guidance [9]) and under greenhouse conditions. It involved the use of four different formulation types (as defined in the catalogue of PPP formulation types and international coding system [10]) (Emulsifiable Concentrate, Suspension Concentrate, Suspoemulsion and Wettable Granule), five distinct compounds featuring stereocentres, which were applied to 11 different crops. In total, the dataset comprises over 1200 data points, of which 366 were below the limit of quantification (LOQ). All available DFR studies conducted by Bayer between 2011 and 2021 where stereospecific measurements were performed were used for the analysis. Studies were excluded from consideration if they did not individually measure all possible isomers. Additionally, studies needed to have at least three DFR trials available for the AS. Finally, a critical distinction was made where isomers need to be considered as metabolites, meaning that not all isomers were present in the initial composition, the concept of the uncertainty factor cannot be applied (uncertainty factor could be infinite), leading to study exclusion, resulting in a final number of 35 DFR studies in the analysis. DFR studies were performed following current guidances [11, 12].

**Fig 1:**
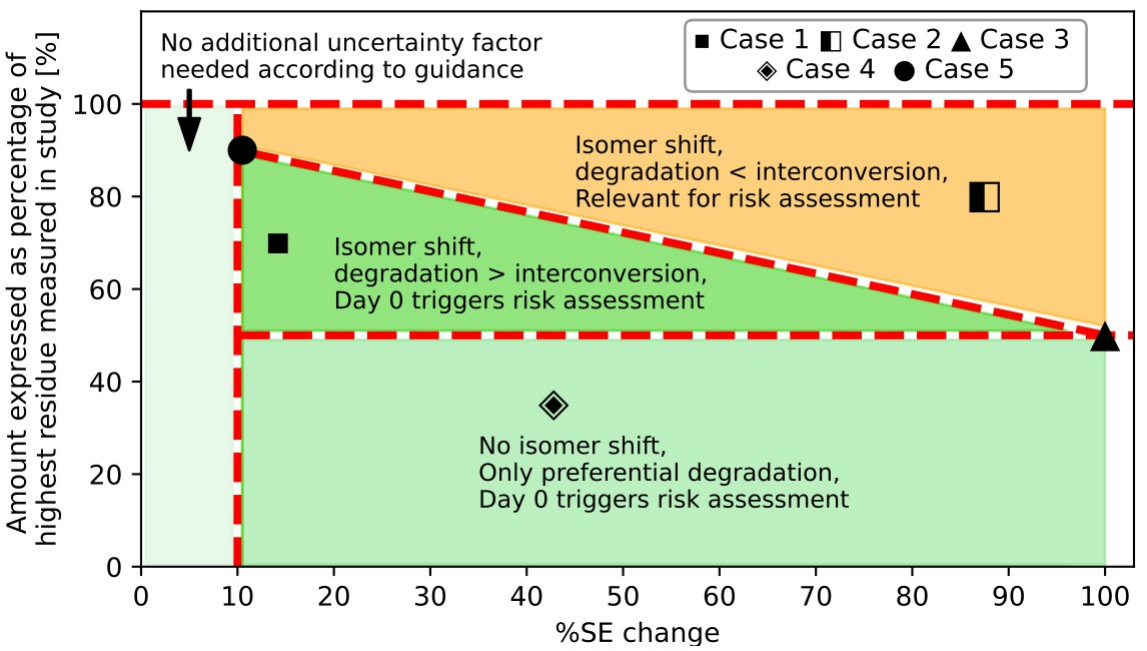
Hypothetical examples from Table 3 showing the combination of % SE change (x-axis) different degradation scenarios (y-axis). SE = Stereoisomeric excess.

**Fig 2:**
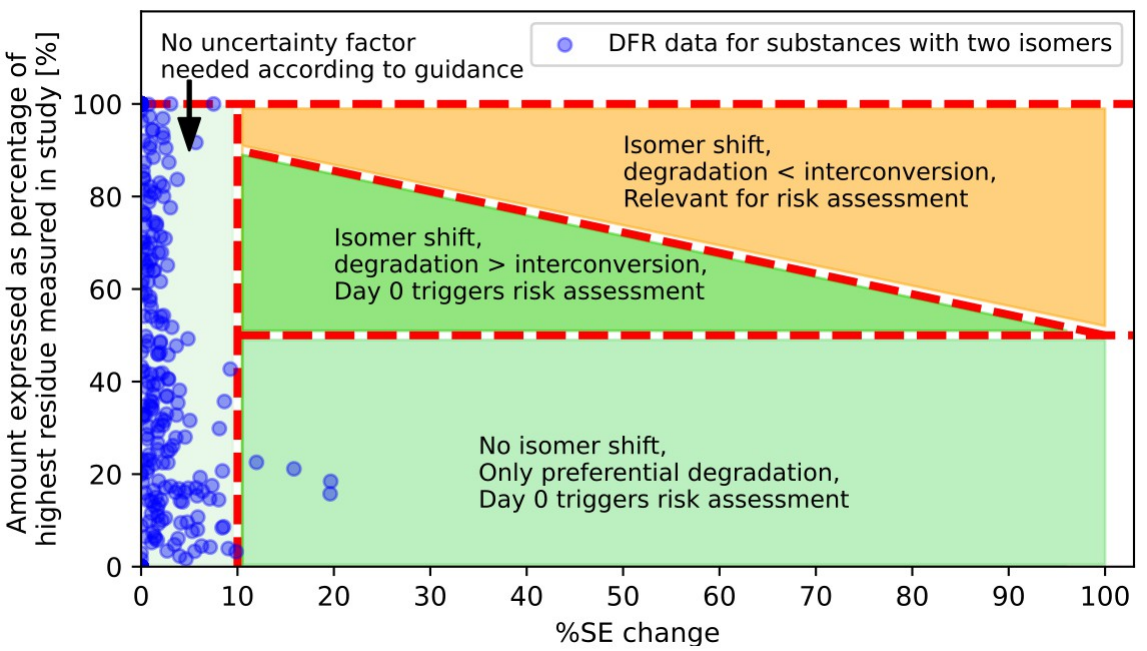
Example including data from four AS with one stereocentre. SE = Stereoisomeric excess.

**Fig 3:**
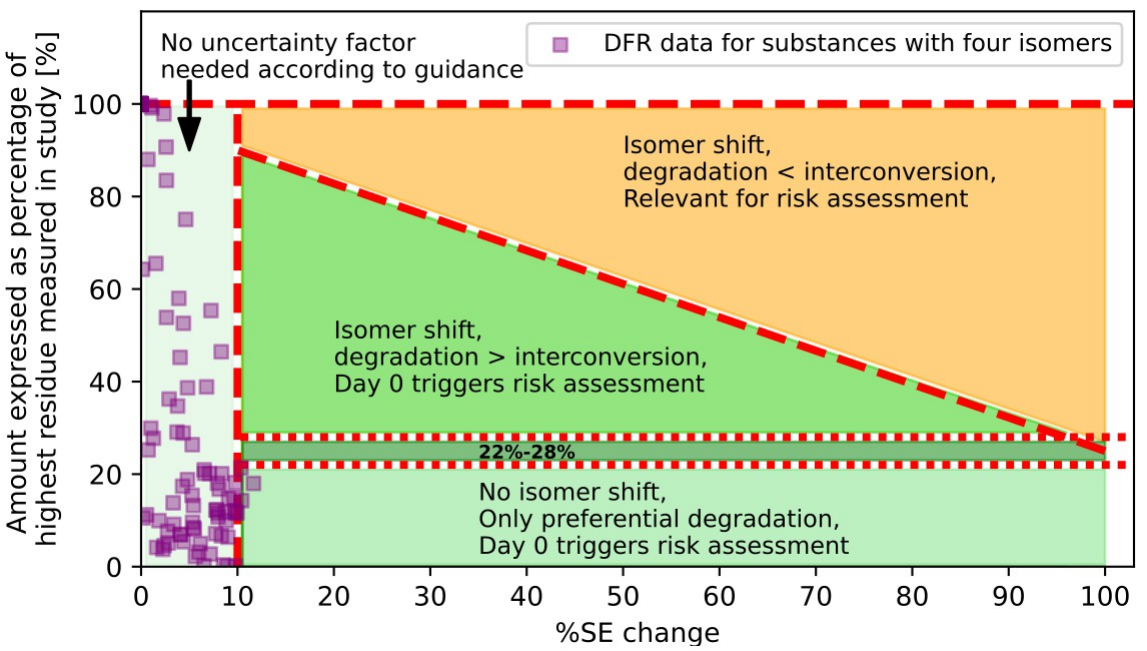
Example for AS comprising of four isomers. SE = Stereoisomeric excess.

**Fig 4:**
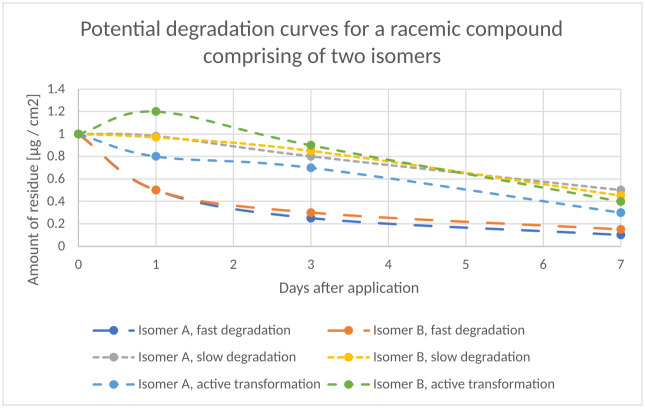
Hypothetical degradation curves of a racemic compound comprising of two isomers.

Figs 1 and 4 show hypothetical data. For Figs 2 and 3, data from these DFR studies were referenced to the point of maximum residue level and its stereoisomeric composition. The maximum residue level was defined as 100%, and the decline in residue was calculated as a percentage of this maximum level. Values were adjusted for field trial recoveries, as outlined in Appendix J of the EFSA Guidance [5]. Single instances are not considered if the guidance allowed to dismiss them (e.g., exceedances where the total amount is below 10% of the initially applied amount, and exceedances which can be excluded as subsequent data do not confirm this exceedance).

## Results and discussion

### Hypothetical considerations

As already mentioned in the introduction, the stereoisomer guidance indicates that an uncertainty factor must be used when the stereoisomeric excess shifts by more than 10% compared to the original composition, usually a racemic mixture, and insufficient information is available to characterise the hazard of the individual stereoisomers. This uncertainty factor is calculated based on the idea that the toxic effects of the isomer mixture can be attributed to a single isomer. If stereospecific measurements are not available, it is assumed that the detected residue consists entirely of this single isomer. This uncertainty factor is then employed to either decrease the toxicological endpoint or increase the relevant residue level, a process applicable to (DFR) studies.

When quantifiable isomer concentrations are available—as is the case for all studies covered in this paper—and the stereoisomeric excess crosses the 10% threshold, it is assumed by following a precautionary approach that the major isomer is solely responsible for the toxic effects. Theoretically, this change in the ratio of isomers can occur by two different processes: either by one isomer degrading faster than the others or by isomerization, which is the active interconversion of one isomer into another.

#### Differences due to different isomer degradation rates

In hypothetical Example 1 an AS with a single stereo centre, composed of two enantiomers in a racemic (i.e., 1:1 or 50%:50%) mixture is considered, whereby only the mixture has been tested in toxicological studies. Thus, there is no toxicological data available for the single isomers (meaning that it remains unknown if the observed toxicity of the racemic mixture comes from both enantiomers equally or if only one enantiomer is responsible for the entire toxicity). If one isomer degrades more quickly, the numbers could hypothetically look like those in Table 1. In this example, we would by default define Isomer B as the more critical isomer since it’s the most abundant. The guidance suggests that only the content of Isomer B would therefore account for the toxicological properties in a hypothetical worst case. The 10% change threshold in stereoisomeric excess is surpassed on the first day after application and the following days. However, if we consider the absolute amount of Isomer B, the amount of Isomer B in the foliar residue never surpasses the Day 0 amount, which is typically the focus in worker risk assessments, because the highest residue level drives the assessment.

**Table 1:** Hypothetical Example 1: AS with one stereo centre, consisting of two enantiomers in a racemic mixture of 1:1, toxicological data available for the racemate but not for the individual enantiomers, preferential degradation. Note that the values for the amount of Isomer A and B are not measured values but are hypothetical and are suggested to illustrate the hypothesis.

Time (days)	Amount Isomer A [μg/cm^2^]	Amount Isomer B [μg/cm^2^]	Rel. amount Isomer A [%]	Rel. amount Isomer B [%]	SE Change^a^ [%]	Relative amount residue^b^ [%]	Amount that needs to be compared with AOEL derived from racemate testing^e^
**0**	0.050	0.050^d^	50	50	0	100	0.1
**1**	0.033	0.048	40.7	59.3	-18.5	81	0.096
**2**	0.015	0.035	30.0	70.0	-40.0	50	0.07
**3**	0.006	0.021	22.2	77.8	-55.6	27	0.042
**5**	<LOQ^c^	0.010	-	-	-	-	
**7**	<LOQ	<LOQ	-	-	-	-	

^a^ SE: Stereoisomeric Excess (Percentage of excess of one stereoisomer over the other in a mixture);

^b^ Relative amount residue: The remaining amount of residue in terms of a percentage relative to the amount at day 0.

^c^ <LOQ = Below the limit of quantification;

^d^ Value relevant for risk assessment;

^e^ If only the racemic mixture of the AS was used to derive the toxicological threshold (Acceptable Operator Exposure level (AOEL)), the residues of the isomer, which is defined most toxic, need to be multiplied by the number of isomers (in this case x 2, if four isomers exist x 4)

The composition of the AS immediately after application is generally understood to maintain the stereoisomer ratio of the PPP, which has been authorized by regulatory agencies. This is primarily because the stability of the formulated PPP is demonstrated in stability studies for its registration. Additionally, as the AS undergoes toxicological studies that provide the data for determining the Acceptable Operator Exposure Level (AOEL), the original ratio of stereoisomers at the time of application is already supported by the toxicological data.

In Hypothetical Example 1, the racemic mixture (a 1:1 ratio for Isomer A and B, both at 0.05 μg/cm²) corresponds to the ratio of isomers used in the toxicological studies. By Day 1 post-application, the stereoisomeric composition necessitates an additional uncertainty factor for Isomer B and simultaneously suspends the exposure by Isomer A. This means that for Day 0 the total exposure is 0.1 μg/ cm^2^ with no additional safety factor, while it is 0.048 (major isomer—Isomer B) and a default safety of 2 for Day 1 (or an uncertainty factor of 1.18 (calculated as 2x [0.048/(0.033+0.048) = 1.18 or 59.3/50 = 1.18]) on the sum of isomers for Day 1, leading to the same result of 0.096 which needs to be compared with the AOEL). It becomes clear that for a racemic mixture with a 1:1 ratio on the day of application a change in the isomeric ratio due to preferential degradation of one isomer does not create a higher risk than the one on Day 0. The assumptions for Hypothetical Example 1 were that toxicological data are available for the racemate only but not for the individual enantiomers, preferential degradation and no interconversion of the two enantiomers takes place and stereoselective measurements are available. If the last point, the stereoselective measurement, is not fulfilled this will lead to a worst-case scenario, i.e., applying a safety factor of 2 on the sum of Isomer A and B even on Day 0. This leads to a hypothetical doubled risk.

#### Differences due to active interconversion

In Hypothetical Example 2 the second possible pathway that could result in a change in the stereoisomeric excess involves active interconversion from one isomer to another. This might occur for an AS with one stereo centre, containing two enantiomers in a racemic mixture of 1:1. A second possible scenario is outlined in Table 2. Isomer A transforms into Isomer B while in parallel degradation takes place. Isomer B would once again be defined as the isomer being responsible for the whole toxicological effect, as it exhibits the highest content (in reality, it’s also possible that Isomer A could be more toxic, or both are equally toxic, but here we consider the worst case). On Day 1 following application, the absolute quantity of Isomer B exceeds the amount initially measured on Day 0. However, by Day 2 post-application, the absolute quantity if isomer B falls below the residue level of Day 0. According to the guidance, only the content of Isomer B contributes to the toxicological hazard, and an additional safety factor of 2 is applied. In this scenario the risk increases from Day 0 to Day 1 and subsequently decreases again. Therefore, applying an additional uncertainty factor in the risk assessment is only relevant on the day of highest concentration of Isomer B–in this example on Day 1. Day 1 would be the worst case in terms of risk through re-entry activities.

**Table 2:** Hypothetical Example 2: AS with one stereocentre, consisting of two enantiomers in a racemic mixture of 1:1. Note that the values for the amount of Isomer A and B are not measured values but are hypothetic and suggested to illustrate the hypothesis.

Time (days)	Amount Isomer A [μg/cm^2^]	Amount Isomer B [μg/cm^2^]	Rel. amount Isomer A [%]	Rel. amount Isomer B [%]	SE Change^a^ [%]	Relative amount residue^b^ [%]	Amount that needs to be compared with AOEL derived from racemate testing
**0**	0.05	0.05	50	50	0	100	0.1
**1**	0.033	0.058^d^	36.3	63.7	-27.5	91	0.116
**2**	0.020	0.037	35.1	64.9	-29.8	57	0.074
**3**	0.006	0.012	33.3	66.7	-33.3	18	0.024
**5**	<LOQ^c^	0.008	-	-	-	-	
**7**	<LOQ	<LOQ	-	-	-	-	

a SE: Stereoisomeric Excess (Percentage of excess of one stereoisomer over the other in a mixture);

b Relative amount residue: The remaining amount of residue in terms of a percentage relative to the amount at day 0;

c <LOQ = Below the limit of quantification;

d Value relevant for risk assessment

#### Simultaneous interconversion and degradation

Interconversion and degradation processes occur simultaneously. Applying the methodology of the guidance, a risk exceeding the one on the day of application is only possible if interconversion is faster than degradation. This situation is illustrated for further cases in Table 3 and Fig 1.

**Table 3:** Hypothetical examples of different degradation scenarios and their impact on %SE change.

	Isomer A	Isomer B	Total amount of residues compared to Day 0	SE change (%)	Exposure to toxic Isomer B
Toxicity	0%	100%			
Residue Day 0	1 mg	1 mg	100%	0%	1 mg
**Changes over time**					
Case 1 (■)	0.6 mg	0.8 mg	70%	14.2%	0.8 mg
Case 2 (◧)	0.1 mg	1.5 mg	80%	87.5%	1.5 mg
Case 3 (▲)	0 mg	1 mg	50%	100%	1 mg
Case 4 (◈)	0.2 mg	0.5 mg	35%	42.8%	0.5 mg
Case 5 (●)	0.8 mg	0.99 mg	90%	10.5%	0.99 mg

Table 3, case 1 (represented by a black square ■ in Fig 1) demonstrates that the quantities of both Isomers A and B decrease, albeit at different rates (averaging 30% decrease compared to the total amount of residue on Day 0), leading to a 14.2% alteration in the stereoisomeric excess. Likewise, cases 3 (black triangle ▲), 4 (white rhombus with black dot ◈), and 5 (black point ●) display degradation of one or both isomers. Conversely, case 2 (black/white square ◧) highlights an isomer interconversion from Isomer A to Isomer B, leading to a higher quantity of Isomer B than the amount measured on Day 0. According to the EFSA guidance, an uncertainty factor must be applied when a change in the stereoisomeric excess surpasses 10%. This is applicable for these hypothetical cases (1–5), even though the absolute amount of the assumed toxic Isomer B exceeds the Day 0 level only in case 2 (◧). At this point, we have no information if the selected cases are also relevant under field condition. A comparison with measured DFR data is provided further below to clarify this question.

Fig 1 illustrates these hypothetical assumptions. Only data points in the upper right orange field of the graph would trigger a worst-case absolute quantity of Isomer B for risk assessment considerations. However, a shift in the isomeric ratio due to preferential degradation of one isomer never impacts the risk assessment. A potential impact on the risk assessment could only occur when an isomer transforms from one to another, and not due to preferential degradation.

#### Compounds with more than one stereocentre

The same considerations are applicable for AS containing more than one stereocentre. The guidance already acknowledges that with an increasing number of isomers, the uncertainty of the approach further escalates. In multi-stereocentral substances, especially in large molecules, the likelihood of degradation or metabolism of the compound, rather than interconversion, is high. Moreover, stereospecific measurements become increasingly complex and the availability of reference substances for all possible isomers can be a limitation. The corresponding figure for an AS with two stereocentres (i.e., four isomers) would look similar, but the horizontal line would be at the 25% mark, assuming an equal distribution of all four isomers (1:1:1:1 ratio in the toxicological studies). This is based on the idea of dividing 1 by the total number of isomers.

### Comparison of measured and hypothetical data

To illustrate how the hypothetical considerations presented in Fig 1 relate to real-world scenarios, we analysed results from 35 set of DFR trials (listed in section 3 of Supplementary Materials). The purpose of this analysis is to demonstrate the practical implications of the theoretical framework, rather than drawing definitive statistical conclusions about the entire population of DFR studies. The analysis includes compounds that display different types of stereogenic elements: Four ASs have a single carbon stereocentre and are racemic mixtures. In addition, one AS was included in the analysis that contained four isomers. The ratio of these isomers in the initial composition was not exactly 1:1:1:1. Therefore, we increased the band area that distinguishes between an isomer shift and no isomer shift to consider this uncertainty.

Fig 2 illustrates the change in stereoisomeric excess (% SE change) on the x-axis for substances with a single stereocentre. According to the guidance, changes up to 10% in enantiomeric excess can be disregarded, and only changes above 10% should be considered. The y-axis represents the percentage of the highest residue measured in the study. The highest residue detected in a study was defined as 100%. For an AS with one stereocentre comprising a 1:1 racemic mixture at the application time, each isomer is present at this time point at 50%. Presuming, as per guidance, that only one isomer contributes to the toxicological effect, all y-axis values below 50% cannot contribute to an increased risk. This is because the absolute amount cannot surpass the amount present at the onset of the study.

The areas highlighted in varying shades of green in the plot will not lead to an increased risk in safety assessments when applying the guidance. Even though there appears to be an interconversion from Isomer A to Isomer B for the four datapoints superseding the 10% SE change threshold no increased risk emerges since the quantity of Isomer A does not surpass the value measured at the beginning of the study. This entire bright green area represents situations where only preferential degradation occurs. In the darker green triangle above, the degradation process is faster than the interconversion process. Only values in the orange area of the plot would result in an increased risk. There were no datapoints in this orange area observed for any of the trials evaluated in this paper. The same scenarios can be used on ASs comprising of four isomers in total. Assuming that all isomers are present in the compound in the same ratio 1:1:1:1 the graph would be adapted with the horizontal line to be at 25% (y-axis, percentage of highest residue measured in study), leading to the same conclusions as above.

In the trials evaluated for this paper data for one compound, being a defined ratio of four isomers in the product, were available. Fig 3 shows the evaluation of this compound. As the ratio of isomers was not exactly 1:1:1:1, the limits between preferential degradation and degradation next to interconversion cannot be assigned as precisely as those in the graphs above but are given as a transition area. Nonetheless, it none of the datapoints land in the orange area.

None of the trials exhibited a scenario where isomerization led to a risk exceeding that observed on the day of application. This observation aligns with our theoretical expectations and suggests that the default uncertainty factors employed according to the EFSA method might be overly conservative. The analysis of this compound serves as an illustrative case study.

### Discussion on an alternative approach to assess risk for workers and residents due to isomerization

As demonstrated above, a scenario relevant to exposure can only occur if interconversion proceeds at a faster rate than degradation. Despite this, the current guidance uncertainty factor for stereoisomers overlooks the time-dependent nature of exposure, and applies a multiplier to the amount of residue, irrespective of the time point. According to the guidance, the approach using uncertainty factor can be extended to include a larger number of isomers. However, the guidance acknowledges that the more isomers that are present, the greater the uncertainty is inherent in the approach. While the guidance suggests the alternative of calculating half-lives and comparing them for mixtures with more isomers, it would be beneficial to include these considerations even for mixtures containing only two isomers to realistically assess the actual risk for workers and residents.

Taking a racemic AS composition as an example, which comprises two isomers in a 1:1 ratio where no hazard information is available for the individual isomers, the endpoint was derived for the racemic mixture. In a worst-case scenario only one of the isomers is responsible for the whole toxicological effect, meaning that the endpoint was derived from only half the dose administered to the test species (the other half being the second isomer which does not account to the toxicological effect at all). To cover this, an uncertainty factor of two should be applied. This uncertainty factor could be taken into account by dividing the toxicological endpoint by two.

Transferring this approach to the cases depicted in Table 3, the toxicological endpoint is divided by a factor of two (because the toxicological endpoint was derived from just half the dose) but is then related to the amount of the major isomer only, being 1 mg. For cases 1, 3, 4 and 5 in Table 3, the risk is not higher than the one directly after application as the amount of the major isomer in these cases decreased. Only in case 2 in Table 3 does the concentration of the major isomer increases relative to the one directly after application as isomerization proceeds faster than degradation. This leads to a higher risk on Day 1 compared to Day 0. Whether isomerization proceeds faster than degradation can readily be derived from DFR study data, in which the residues are monitored by sampling several days after application. Potential degradation curves of DFR are shown in Fig 4.

For an AS comprising two isomers which are both declining very quickly, already one day after application, a strong decline of the residue can be observed. In this example, both isomers decline and there is no active interconversion from one isomer into the other. In case of a stagnation and slow decline of the isomers, which is outlined in Fig 4 for Isomers A and B with slow degradation, again no active interconversion from one isomer into the other can be observed.

A scenario leading to a higher risk compared to Day 0 would only occur if one of the curves superseded the measured amount on Day 0 on the y-axis (curve Isomer B, active interconversion). In none of the studies evaluated for this paper, datapoints indicating faster interconversion than degradation that led to higher risk compared to Day 0 were observed. All available data indicate that degradation is faster than interconversion of stereoisomers. Therefore, no higher risk compared to day 0 can be derived. Simplifying the graphs to formulas, it can be derived:

$$If\;{Slope}_{Isomerization}<{Slope}_{Degradation}$$

or

$$if\;{Absolute\;residue}_{\;IsomerX\;DayY\;}\;\leq {Absolute\;residue}_{IsomerX\;at\;day\;of\;application}$$

(with Isomer X being the isomer with the higher residue and by that, according to guidance, the isomer considered to be responsible for the whole toxicological effect) no higher risk compared to the day of application can be derived and an uncertainty factor is not justified. This approach and conclusion described above are based on the availability of analytical data of stereoisomers. In case only data for the sum of isomers are available no conclusion about isomer interconversion is possible, but about degradation it is. If soon after application (e.g., after one day) a residue below 50% of the initial residue is found, the degradation process is considered dominant. Consequently, in such cases no higher risk compared to the time of application is to be expected.

## Conclusions

Studies assessing real exposure scenarios are crucial for accurate risk assessment. The EFSA stereoisomer guidance recommends considering uncertainty factors to account for possible isomerization with regards to non-dietary exposure. Our data demonstrate that relevant DFR studies conducted by Bayer, representing the relevant exposure scenario for groups that may be impacted by changes in stereoisomeric composition, can serve as surrogates to cover re-entry and surface deposit scenarios for PPPs.

The results of our evaluation indicate how to adjust the evaluation of stereoisomeric composition changes. Field studies have higher variation and a faster decline in foliar residues compared to pure laboratory trials, making it challenging to fully apply the measures defined in the EFSA guidance to DFR studies. Moreover, ruling out exceedance values using subsequent data becomes difficult due to the rapid decline of residues. As a result, an alternative assessment is required to address realistic non-dietary exposure scenarios.

Our findings underscore the importance of utilizing empirical data from DFR studies to calculate exposure and cover the associated risks as the decline of residues on the leaf surface must be considered. The example calculations here emphasize the significance of considering the absolute amount of each isomer on the leaf surface when assessing risk. It is essential to note that exceeding the 10% threshold for stereoisomeric excess change does not necessarily indicate exposure to a significant amount of an isomer not covered by toxicological studies. A relevant exposure scenario arises only when the isomerization rate is faster than the degradation of the compound on the leaf surface. Therefore, the threshold of 10% SE change defined by EFSA could be replaced by evaluation of degradation versus isomerization rate in DFR data for assessing non-dietary risk.

It is acknowledged that our analysis of DFR studies serves as an illustrative case study, demonstrating the alignment between empirical data and the theoretical concerns regarding the EFSA method. This empirical analysis, derived from real-world field studies with stereoselective measurements, supports our hypothesis that preferential degradation alone does not necessitate an additional safety factor. Our dataset revealed that there were no instances where isomerization led to a higher risk compared to the day of application. Although we have evaluated data from 35 studies over a ten-year period, the database is still limited. To claim representativity for the full spectrum of PPPs more data would need to be generated. Especially assessing the exposure and risk for AS with a high number of isomers remains problematic, necessitating further investigations to better understand the actual risks.

It should also be considered that our approach is only applicable for ASs that contain stereoisomers already in their composition. For ASs containing only one stereoisomer among those possibly formed on the basis of the AS molecular structure (and/or if other isomers are present, they are considered amount-wise to be impurities), we propose to treat other stereoisomers formed as metabolites.

Further research, including comparisons of measured DFR data, is needed to determine if observations in this study also apply to DFR studies from other sources.

## Supporting information

S1 File Glossary (of terminology used in the manuscript), materials and methods (containing information on the conduct of dislodgeable foliar residue studies), raw data used for Figs 2 and 3. (DOCX)
