# Supplementary material for: Use of dislodgeable foliar residue studies for the evaluation of isomerization potential of active substances for non-dietary risk assessment
Source: PLoS One. 2024 Nov 1;19(11):e0312688. doi: 10.1371/journal.pone.0312688 (PMC11530052; doi:10.1371/journal.pone.0312688)
Supplement: S1 File — (DOCX) [file pone.0312688.s001.docx]

**Supplementary Materials**

**1 Glossary**

- **Acceptable Operator Exposure Level (AOEL):** The maximum amount of active substance to which an operator can be exposed without the risk of experiencing adverse health effects.
- **Bystander:** A person who is present near a treated area during or shortly after pesticide application, potentially exposed to spray drift or volatilized pesticide.
- **Dislodgeable Foliar Residue (DFR) Study:** A study that measures the amount of pesticide residue that can be removed from plant leaves or other surfaces by contact, simulating potential exposure for workers or residents.
- **EFSA (European Food Safety Authority):** The European Union agency responsible for assessing risks associated with food and feed safety, including the evaluation of pesticides and their potential impact on human health and the environment.
- **Emulsifiable Concentrate, Suspension Concentrate, Suspoemulsion, and Wettable Granule:** Different types of pesticide formulations.
- **Enantioselective degradation:** The preferential degradation of one enantiomer over the other.
- **Environmental Fate Study:** An investigation of the behavior of a pesticide in the environment, including its degradation, transport, and accumulation in various compartments like soil, water, and air.
- **GLP (Good Laboratory Practice):** A quality system concerned with the organizational process and the conditions under which non-clinical health and environmental safety studies are planned, performed, monitored, recorded, archived and reported.
- **Higher tier assessment:** A more refined risk assessment that uses additional data and models to estimate exposure and risk.
- **Interconversion:** The transformation of one isomer into another.
- **Northern and southern residue zones:** Geographical regions in Europe with different climatic conditions that can affect pesticide residues.
- **Operator:** The person who handles and applies the pesticide.
- **Racemic mixture:** A mixture containing equal amounts of the respective isomers.
- **Re-entry Scenario:** The situation where workers or residents re-enter a treated field after pesticide application, potentially exposing them to residues on crops or in the soil.
- **Residue Study:** A study that determines the amount of pesticide residue remaining in or on treated crops or livestock over time, ensuring that residues do not exceed safe levels for consumption.
- **Resident:** An individual living near an area where pesticides are applied, with potential for long-term exposure to residues through various pathways like air, water, or soil.
- **Stereogenic element/centre:** The structural moiety of a molecule that gives rise to stereoisomers.
- **Stereoisomeric excess (SE):** The excess of relative amount of one isomer over the other in a mixture.
- **Toxicological endpoint:**  Systemic endpoint normally based on a sub-chronic tox study conducted in animals, derived from the no adverse effect level, including safety factors and correction for absorption.
- **Uncertainty factor:** A numerical factor used in risk assessment to account for uncertainties in the data or assumptions.
- **Worker:** An individual who performs tasks in a treated field after pesticide application, such as harvesting or pruning, with potential exposure to residues.

**2 Materials and methods**

**2.1 Conduct of dislodgeable foliar residue (DFR) studies**

**2.1.1 Plot design**

The plot was divided into three sub-plots for sampling. Untreated control samples as well as all samples needed for the field recoveries were collected prior to the first application of the test item.

**2.1.2 Collecting of leaf punches**

Samples were collected in a manner designed to obtain representative samples. They were taken, prepared in the field where necessary, transported and stored according to US EPA OPPTS 875.2100 Foliar Dislodgeable Residue Dissipation. Leaf punches were collected directly into a pre-labelled poly-propylene jar using a leaf punch sampler.

The leaf puncher diameter was chosen depending on the crop sampled. For punchers with a 2.523 cm diameter, 40 punches were collected, resulting in a total of 400 cm^2^ collected. For punchers with a 1.784 or 1.262 cm diameter, 80 punches were collected, resulting in a total area of 400 cm^2^ or 200 cm^2^, respectively. A sample was collected from each of the three subplots to provide three replicates at each sampling interval. Leaf punches were taken randomly over the inner parts of the subplots from the potential worker contact zone. Control leaf punch samples were collected prior to the first application. Treated samples collected on the day of application were taken after the spray had dried. After each sample was collected, the sampling jar was capped and transported to the field site laboratory for dislodging. Leaf punch samplers were cleaned after each sampling interval.

**2.1.3 Dislodgeable foliar residue sample collection**

The dislodging of the leaf samples was performed as soon as possible, but not later than 4 hours after collection, by adding 100 mL of a 0.01 % Aerosol OT solution into the jar containing the leaf material. The jar was shaken for ten minutes at approximately 200 cycles per minute. The solution was decanted. The dislodging procedure was repeated for each sample with a fresh 100 mL aliquot of the Aerosol solution. The second rinse was again decanted and added to the first. The samples were deep frozen, and the dislodged leaves were discarded.

**2.1.4 Control and field recovery samples**

Field fortification samples were used to demonstrate the stability of the samples during storage period of the study and the ability of the analytical laboratory to recover an analyte fortified into a sample at the field test site. The solutions from control samples were fortified with the respective active substances at the LOQ and at a higher concentration level.

**2.1.5 Analysis**

The analysis of the dislodging solutions was performed with methods specifically developed and validated for the respective active substances. The validations were performed under GLP testing the parameters selectivity, linearity, limit of quantification, accuracy, repeatability, limit of detection and stability of the solutions. Quantification involved the use of isotopically stable labelled internal standards and MS/MS detection.

Quantification of enantiomers evolved during the ten years period over which the studies included in this paper were performed. For the compound containing four isomers, the first studies included only the measurements of the diastereomers (marked in this supplemental information). For the compounds with a single carbon stereocentre in the first studies the extracts of quantification were analysed a second time using reversed phase chromatography on a chiral HPLC column to obtain the ratio of enantiomers. Subsequently, methods were specifically validated for the single enantiomers and the single enantiomers were quantified.

**2.2 Evaluation of DFR data according to EFSA stereoisomer guidance**

Data from DFR studies were evaluated by:

1. Correcting residues on the leaf surfaces with field trial recoveries of the respective study. In the majority of studies, field trial recoveries for each isomer were available. In some older studies only a value for the sum of isomers was available; however, no correction of the values was performed as this would not have any effect on the result.
2. Calculation of the molar fractions for each isomer using the following formula (according to EFSA stereoisomer guidance, Appendix A) for active substances comprising of two isomers (called Isomer 1 and 2):

$$Molar fraction Isomer 1= \frac{Residue (Isomer 1)}{Residue \left( Isomer 1 \right)+Residue (Isomer 2)}*100\%$$

$Molar fraction Isomer 2= \frac{Residue (Isomer 2)}{Residue \left( Isomer 1 \right)+Residue (Isomer 2)}*100$%

Stereoisomeric excess (SE):

$$SE \left( \% \right)=Molar fraction \left( Isomer 1 \right)-Molar fraction (Isomer 2)$$

Change of stereoisomeric excess:

$$SE change\left( \% \right)= {SE}_{Day with highest residue level}-{SE}_{Timepoint X}$$

For the active substance comprising of four, which is a defined ratio of diastereomers, (Diastereomer 1 being the sum of Isomer 1 and 2, and Diastereomer 2 being the sum of Isomer 3 and 4), the following formula was applied:

$$Molar fraction Diastereomer 1= \frac{Residue \left( Isomer 1 \right)+Residue (Isomer 2)}{Residue \left( Isomer 1 \right)+Residue \left( Isomer 2 \right)+Residue \left( Isomer 3 \right)+Residue (Isomer 4)}*100\%$$

$$Molar fraction Diastereomer 2= \frac{Residue \left( Isomer 3 \right)+Residue (Isomer 4)}{Residue \left( Isomer 1 \right)+Residue \left( Isomer 2 \right)+Residue \left( Isomer 3 \right)+Residue (Isomer 4)}*100\%$$

$$SE \left( \% \right)=Molar fraction \left( Diastereomer 1 \right)-Molar fraction (Diastereomer 2)$$

1. Defining the highest residue level measured (as sum of isomers) within the DFR study as 100% and correlating the dislodgeable foliar residues from further sampling events to this highest residue level.
2. Generating graphs by correlating the % SE change to the amount expressed as percentage of highest residue measured in study.
3. Disregard exceedances of the 10% SE change threshold if the amount expressed as percentage of highest residue measured in study is lower than 10% (which is in-line with the description of EFSA stereoisomer guidance Appendix A acknowledging that errors in the analytical measurements might lead to an exceedance of the 10% SE change threshold if the residues measured are very low. If the amount of residue is below 10% of the original applied dose (which is, in case of the DFR study, the highest residue measured), this exceedance of the threshold has low relevance).
4. Disregard exceedances of the 10% SE change threshold if subsequent data points do not confirm this exceedance and indicate that the superseding is due to experimental error.

**3** **Supplementary data used for Figure 2 (compound 1, 2, 3 and 4) and for Figure 3 (compound 5).** DFR data for 5 compounds, each tested in up to 18 studies. As some compounds were tested within the same experiment/study, in total 35 studies were performed.

<LoQ: residues measured were below the Limit of Quantification (LOQ). No calculation of molar fractions and SE possible

*Disregarded value in graph as described in supplemental information “evaluation of DFR data according to EFSA stereoisomer guidance” under point 5

** Disregarded value in graph as described in supplemental information “evaluation of DFR data according to EFSA stereoisomer guidance” under point 6

**Compound 1**

| Study | DAF | Molar fraction Isomer 1  [%] | Molar fraction Isomer 2  [%] | Stereoisomeric excess (SE) [%] | SE change [%] | | Amount expressed as percentage of highest residue measured in study [%] |
| --- | --- | --- | --- | --- | --- | --- | --- |
| 1 | 0 | 50,2 | 49,8 | 0,4 | 1,6 | | 74 |
|  | 10/-0 | 49,1 | 50,9 | -1,7 | 3,7 | | 33 |
|  | 0 | 51,0 | 49,0 | 2,0 | 0,0 | | 100 |
|  | 1 | 49,5 | 50,5 | -1,1 | 3,0 | | 78 |
|  | 3 | 49,7 | 50,3 | -0,7 | 2,6 | | 33 |
|  | 7 | 48,7 | 51,3 | -2,6 | 4,6 | | 28 |
|  | 10 | 49,9 | 50,1 | -0,2 | 2,1 | | 32 |
| 2 | 0 | 49,9 | 50,1 | -0,1 | 1,8 | | 68 |
|  | 7/-0 | <LoQ | <LoQ | <LoQ | <LoQ | | <LoQ |
|  | 0 | 50,2 | 49,8 | 0,4 | 1,3 | | 64 |
|  | 7/-0 | <LoQ | <LoQ | <LoQ | <LoQ | | <LoQ |
|  | 0 | 50,8 | 49,2 | 1,7 | 0,0 | | 100 |
|  | 1 | 50,9 | 49,1 | 1,8 | -0,1 | | 59 |
|  | 3 | 51,8 | 48,2 | 3,7 | -2,0 | | 48 |
|  | 7 | 51,7 | 48,3 | 3,4 | -1,7 | | 40 |
|  | 10 | <LoQ | <LoQ | <LoQ | <LoQ | | <LoQ |
| 3 | 0 | 51,4 | 48,6 | 2,8 | -2,5 | | 71 |
|  | 7/-0 | <LoQ | <LoQ | <LoQ | <LoQ | | <LoQ |
|  | 0 | 50,2 | 49,8 | 0,3 | 0,0 | | 100 |
|  | 7/-0 | 49,9 | 50,1 | -0,3 | 0,6 | | 39 |
|  | 0 | 50,6 | 49,4 | 1,3 | -0,9 | | 80 |
|  | 1 | 49,9 | 50,1 | -0,1 | 0,5 | | 50 |
|  | 3 | 51,6 | 48,4 | 3,1 | -2,8 | | 46 |
|  | 7 | 49,2 | 50,8 | -1,5 | 1,9 | | 42 |
|  | 11 | 51,5 | 48,5 | 3,0 | -2,7 | | 37 |
| 4 | 0 | 50,7 | 49,3 | 1,5 | -0,7 | | 64 |
|  | 7/-0 | 50,9 | 49,1 | 1,8 | -1,0 | | 36 |
|  | 0 | 50,4 | 49,6 | 0,8 | 0,0 | | 100 |
|  | 7/-0 | <LoQ | <LoQ | <LoQ | <LoQ | | <LoQ |
|  | 0 | 50,9 | 49,1 | 1,7 | -0,9 | 91 | |
|  | 1 | 49,3 | 50,7 | -1,3 | 2,1 | 68 | |
|  | 3 | 49,3 | 50,7 | -1,4 | 2,2 | 42 | |
|  | 7 | <LoQ | <LoQ | <LoQ | <LoQ | <LoQ | |
|  | 10 | <LoQ | <LoQ | <LoQ | <LoQ | <LoQ | |
| 5 | 0 | 50,3 | 49,7 | 0,6 | 2,0 | 71 | |
|  | 7/-0 | <LoQ | <LoQ | <LoQ | <LoQ | <LoQ | |
|  | 0 | <LoQ | <LoQ | <LoQ | <LoQ | <LoQ | |
|  | 7/-0 | <LoQ | <LoQ | <LoQ | <LoQ | <LoQ | |
|  | 0 | 51,3 | 48,7 | 2,6 | 0,0 | 100 | |
|  | 1 | 52,2 | 47,8 | 4,4 | -1,8 | 46 | |
|  | 3 | 52,0 | 48,0 | 3,9 | -1,3 | 37 | |
|  | 7 | <LoQ | <LoQ | <LoQ | <LoQ | <LoQ | |
|  | 10 | <LoQ | <LoQ | <LoQ | <LoQ | <LoQ | |
| 6 | 0 | 50,9 | 49,1 | 1,9 | 2,3 | 93 | |
|  | 7/-0 | <LoQ | <LoQ | <LoQ | <LoQ | <LoQ | |
|  | 0 | 50,2 | 49,8 | 0,4 | 3,8 | 84 | |
|  | 7/-0 | 50,9 | 49,1 | 1,8 | 2,4 | 32 | |
|  | 0 | 52,1 | 47,9 | 4,2 | 0,0 | 100 | |
|  | 1 | 49,2 | 50,8 | -1,5 | 5,7 | 92 | |
|  | 3 | 47,8 | 52,2 | -4,5 | 8,6 | 36 | |
|  | 7 | 50,7 | 49,3 | 1,4 | 2,8 | 41 | |
|  | 10 | 50,1 | 49,9 | 0,2 | 4,0 | 38 | |
| 7 | 0 | 50,3 | 49,7 | 0,7 | 0,0 | 100 | |
|  | 7 | <LoQ | <LoQ | <LoQ | <LoQ | <LoQ | |
|  | 0 | 51,2 | 48,8 | 2,4 | -1,8 | 46 | |
|  | 7 | <LoQ | <LoQ | <LoQ | <LoQ | <LoQ | |
|  | 0 | 50,8 | 49,2 | 1,6 | -0,9 | 81 | |
|  | 1 | 49,8 | 50,2 | -0,4 | 1,0 | 70 | |
|  | 3 | 48,7 | 51,3 | -2,5 | 3,2 | 52 | |
|  | 7 | <LoQ | <LoQ | <LoQ | <LoQ | <LoQ | |
|  | 10 | <LoQ | <LoQ | <LoQ | <LoQ | <LoQ | |
| 8 | 0 | 50,9 | 49,1 | 1,8 | 1,2 | 94 | |
|  | 10/-0 | <LoQ | <LoQ | <LoQ | <LoQ | <LoQ | |
|  | 0 | 51,5 | 48,5 | 3,0 | 0,0 | 100 | |
|  | 1 | 50,9 | 49,1 | 1,8 | 1,2 | 94 | |
|  | 3 | 50,5 | 49,5 | 1,0 | 2,1 | 54 | |
|  | 7 | 51,1 | 48,9 | 2,1 | 0,9 | 35 | |
|  | 10 | 52,9 | 47,1 | 5,9 | -2,9 | 40 | |
| 9 | 0 | 50,8 | 49,2 | 1,5 | 0,7 | 69 | |
|  | 7 | <LoQ | <LoQ | <LoQ | <LoQ | <LoQ | |
|  | 14 | <LoQ | <LoQ | <LoQ | <LoQ | <LoQ | |
|  | 0 | 51,1 | 48,9 | 2,3 | 0,0 | 100 | |
|  | 7 | <LoQ | <LoQ | <LoQ | <LoQ | <LoQ | |
|  | 12 | <LoQ | <LoQ | <LoQ | <LoQ | <LoQ | |
|  | 0 | 50,7 | 49,3 | 1,4 | 0,9 | 97 | |
|  | 1 | 50,0 | 50,0 | 0,0 | 2,3 | 97 | |
|  | 3 | 52,1 | 47,9 | 4,2 | -1,9 | 83 | |
|  | 8 | 51,4 | 48,6 | 2,7 | -0,5 | 48 | |
|  | 9 | 50,1 | 49,9 | 0,3 | 2,0 | 46 | |

**Compound 2**

| Study | DAF | Molar fraction Isomer 1  [%] | Molar fraction Isomer 2  [%] | Stereoisomeric excess (SE) [%] | SE change [%] | | Amount expressed as percentage of highest residue measured in study [%] |
| --- | --- | --- | --- | --- | --- | --- | --- |
| 1 | 0 | 49,9 | 50,1 | -0,3 | 0 | | 100 |
|  | 1 | 50,0 | 50,0 | 0,1 | -0,4 | | 87 |
|  | 3 | 50,0 | 50,0 | -0,1 | -0,2 | | 85 |
|  | 7 | 49,7 | 50,3 | -0,6 | 0,4 | | 6 |
|  | 14/-0 | 49,8 | 50,2 | -0,3 | 0,0 | | 61 |
|  | 1 | 49,6 | 50,4 | -0,9 | 0,6 | | 29 |
|  | 3 | 49,4 | 50,6 | -1,1 | 0,8 | | 24 |
|  | 7 | 48,1 | 51,9 | -3,7 | 3,5 | | 5 |
| 2 | 0 | 49,9 | 50,1 | -0,2 | 0,8 | | 100 |
|  | 1 | 50,0 | 50,0 | 0,0 | 0,6 | | 23 |
|  | 3 | 50,0 | 50,0 | -0,1 | 0,7 | | 21 |
|  | 7 | 50,1 | 49,9 | 0,2 | 0,4 | | 16 |
|  | 10 | 49,9 | 50,1 | -0,2 | 0,9 | | 23 |
|  | 14/-0 | 49,7 | 50,3 | -0,6 | 1,2 | | 8 |
|  | 0 | 50,3 | 49,7 | 0,6 | 0,0 | | 100 |
|  | 1 | 49,7 | 50,3 | -0,7 | 1,3 | | 74 |
|  | 3 | 49,8 | 50,2 | -0,4 | 1,0 | | 42 |
|  | 7 | 49,7 | 50,3 | -0,7 | 1,3 | | 16 |
|  | 10 | 50,0 | 50,0 | -0,1 | 0,7 | | 14 |
| 3 | 0 | 48,7 | 51,3 | -2,7 | 0 | | 100 |
|  | 1 | 48,3 | 51,7 | -3,4 | 0,7 | | 61 |
|  | 3 | 48,1 | 51,9 | -3,9 | 1,2 | | 36 |
|  | 7 | 47,3 | 52,7 | -5,4 | 2,7 | | 25 |
|  | 10 | 47,1 | 52,9 | -5,7 | 3,0 | | 25 |
|  | 14/-0 | 44,6 | 55,4 | -10,7 | 8,0 | | 14 |
|  | 0 | 48,0 | 52,0 | -3,9 | 1,3 | | 88 |
|  | 1 | 47,5 | 52,5 | -5,0 | 2,3 | | 54 |
|  | 3 | 48,0 | 52,0 | -4,0 | 1,3 | | 39 |
|  | 7 | 47,0 | 53,0 | -6,0 | 3,3 | | 26 |
|  | 10 | 45,8 | 54,2 | -8,4 | 5,7 | 17 | |

**Compound 3**

| Study | DAF | Molar fraction Isomer 1  [%] | Molar fraction Isomer 2  [%] | Stereoisomeric excess (SE) [%] | SE change [%] | | Amount expressed as percentage of highest residue measured in study [%] |
| --- | --- | --- | --- | --- | --- | --- | --- |
| 1 | 0 | 50,0 | 50,0 | 0,0 | 0 | | 100 |
|  | 1 | 49,7 | 50,3 | -0,7 | 0,7 | | 28 |
|  | 3 | 50,7 | 49,3 | 1,4 | -1,4 | | 7 |
|  | 7 | 52,8 | 47,2 | 5,6 | -5,6 | | 3 |
|  | 10 | 50,0 | 50,0 | 0,0 | 0,0 | | 3 |
|  | 14 | 50,0 | 50,0 | 0,0 | 0,0 | | 2 |
|  | 0 | 49,9 | 50,1 | -0,1 | 0,1 | | 62 |
|  | 1 | 50,0 | 50,0 | 0,0 | 0,0 | | 20 |
|  | 3 | 50,0 | 50,0 | 0,0 | 0,0 | | 9 |
|  | 7 | 51,4 | 48,6 | 2,7 | -2,7 | | 3 |
|  | 10 | 52,0 | 48,0 | 4,0 | -4,0 | | 2 |
| 2 | 0 | 49,9 | 50,1 | -0,2 | 0 | | 100 |
|  | 1 | 50,2 | 49,8 | 0,5 | -0,7 | | 49 |
|  | 0 | 49,6 | 50,4 | -0,7 | 0,5 | | 33 |
| 3 | 0 | 50,1 | 49,9 | 0,1 | -2,7 | | 65 |
|  | 1 | 49,2 | 50,8 | -1,5 | -1,1 | | 5 |
|  | 10 | 50,0 | 50,0 | 0,0 | -2,6 | | 90 |
|  | 0 | 48,7 | 51,3 | -2,6 | 0,0 | | 100 |
|  | 1 | 51,1 | 48,9 | 2,2 | -4,9 | | 49 |
|  | 3 | 50,1 | 49,9 | 0,2 | -2,8 | | 22 |
| 4 | 0 | 47,1 | 52,9 | -5,8 | 0 | | 100 |
|  | 1 | 51,6 | 48,4 | 3,2 | -9,0 | | 4 |
|  | 14 | <LoQ | <LoQ | <LoQ | <LoQ | |  |
|  | 0 | 48,2 | 51,8 | -3,6 | -2,2 | | 52 |
|  | 1 | <LoQ | <LoQ | <LoQ | <LoQ | |  |
|  | 3 | <LoQ | <LoQ | <LoQ | <LoQ | |  |
|  | 7 | <LoQ | <LoQ | <LoQ | <LoQ | |  |
|  | 10 | <LoQ | <LoQ | <LoQ | <LoQ | |  |
| 5 | 0 | 50,6 | 49,4 | 1,2 | -1,2 | | 71 |
|  | 1 | 50,0 | 50,0 | 0,0 | 0,0 | 100 | |
|  | 14 | <LoQ | <LoQ | <LoQ | <LoQ |  | |
|  | 0 | 51,1 | 48,9 | 2,2 | -2,2 | 94 | |
|  | 1 | 51,4 | 48,6 | 2,9 | -2,9 | 87 | |
|  | 3 | <LoQ | <LoQ | <LoQ | <LoQ |  | |
|  | 7 | <LoQ | <LoQ | <LoQ | <LoQ |  | |
|  | 9 | <LoQ | <LoQ | <LoQ | <LoQ |  | |
| 6 | 0 | 49,6 | 50,4 | -0,8 | 0 | 100 | |
|  | 1 | 50,5 | 49,5 | 0,9 | -1,8 | 29 | |
|  | 14 | <LoQ | <LoQ | <LoQ | <LoQ |  | |
|  | 0 | 49,7 | 50,3 | -0,6 | -0,2 | 69 | |
|  | 1 | 50,0 | 50,0 | 0,0 | -0,8 | 30 | |
|  | 3 | <LoQ | <LoQ | <LoQ | <LoQ |  | |
|  | 7 | <LoQ | <LoQ | <LoQ | <LoQ |  | |
|  | 9 | <LoQ | <LoQ | <LoQ | <LoQ |  | |
| 7 | 0 | 50,4 | 49,6 | 0,9 | 0 | 100 | |
|  | 1 | 51,3 | 48,7 | 2,6 | -1,7 | 6 | |
|  | 2 | <LoQ | <LoQ | <LoQ | <LoQ |  | |
|  | 8 | <LoQ | <LoQ | <LoQ | <LoQ |  | |
|  | 9 | <LoQ | <LoQ | <LoQ | <LoQ |  | |
| 8 | 0 | 49,0 | 51,0 | -2,0 | -0,1 | 96 | |
|  | 1 | <LoQ | <LoQ | <LoQ | <LoQ |  | |
|  | 3 | <LoQ | <LoQ | <LoQ | <LoQ |  | |
|  | 7 | <LoQ | <LoQ | <LoQ | <LoQ |  | |
|  | 10 | <LoQ | <LoQ | <LoQ | <LoQ |  | |
|  | 0 | 49,0 | 51,0 | -2,0 | 0,0 | 100 | |
|  | 1 | <LoQ | <LoQ | <LoQ | <LoQ |  | |
|  | 3 | <LoQ | <LoQ | <LoQ | <LoQ |  | |
|  | 7 | <LoQ | <LoQ | <LoQ | <LoQ |  | |
|  | 10 | <LoQ | <LoQ | <LoQ | <LoQ |  | |
|  | 0 | 49,4 | 50,6 | -1,1 | -0,9 | 90 | |
|  | 1 | 47,7 | 52,3 | -4,5 | 2,5 | 17 | |
|  | 3 | <LoQ | <LoQ | <LoQ | <LoQ |  | |
|  | 5 | <LoQ | <LoQ | <LoQ | <LoQ |  | |
|  | 7 | <LoQ | <LoQ | <LoQ | <LoQ |  | |
|  | 10 | <LoQ | <LoQ | <LoQ | <LoQ |  | |
| 9 | 0 | 51,4 | 48,6 | 2,9 | -2,3 | 80 | |
|  | 1 | 51,1 | 48,9 | 2,2 | -1,7 | 12 | |
|  | 3 | <LoQ | <LoQ | <LoQ | <LoQ |  | |
|  | 10 | <LoQ | <LoQ | <LoQ | <LoQ |  | |
|  | 0 | 50,3 | 49,7 | 0,6 | 0,0 | 100 | |
|  | 1 | <LoQ | <LoQ | <LoQ | <LoQ |  | |
|  | 3 | <LoQ | <LoQ | <LoQ | <LoQ |  | |
|  | 10 | <LoQ | <LoQ | <LoQ | <LoQ |  | |
|  | 0 | 50,1 | 49,9 | 0,3 | 0,3 | 84 | |
|  | 1 | 52,7 | 47,3 | 5,4 | -4,9 | 17 | |
|  | 3 | <LoQ | <LoQ | <LoQ | <LoQ |  | |
|  | 5 | <LoQ | <LoQ | <LoQ | <LoQ |  | |
|  | 7 | <LoQ | <LoQ | <LoQ | <LoQ |  | |
|  | 10 | <LoQ | <LoQ | <LoQ | <LoQ |  | |

| Study | DAF | Molar fraction Isomer 1  [%] | Molar fraction Isomer 2  [%] | Stereoisomeric excess (SE) [%] | SE change [%] | | Amount expressed as percentage of highest residue measured in study [%] |
| --- | --- | --- | --- | --- | --- | --- | --- |
| 10 | 0 | 48,9 | 51,1 | -2,2 | 0,9 | | 58 |
|  | 21 | <LoQ | <LoQ | <LoQ | <LoQ | |  |
|  | 0 | 49,4 | 50,6 | -1,3 | 0,0 | | 100 |
|  | 1 | 48,8 | 51,2 | -2,4 | 1,1 | | 12 |
|  | 3 | <LoQ | <LoQ | <LoQ | <LoQ | |  |
|  | 7 | <LoQ | <LoQ | <LoQ | <LoQ | |  |
| 11 | 0 | 52,9 | 47,1 | 5,9 | 0 | | 100 |
|  | 1 | 47,0 | 53,0 | -6,1 | 12,0 | | 23 |
|  | 3 | <LoQ | <LoQ | <LoQ | <LoQ | |  |
|  | 7 | <LoQ | <LoQ | <LoQ | <LoQ | |  |
|  | 10 | <LoQ | <LoQ | <LoQ | <LoQ | |  |
| 12 | 0 | 50,9 | 49,1 | 1,7 | -0,9 | | 57 |
|  | 1 | 51,1 | 48,9 | 2,3 | -1,5 | | 6 |
|  | 3 | <LoQ | <LoQ | <LoQ | <LoQ | |  |
|  | 7 | <LoQ | <LoQ | <LoQ | <LoQ | |  |
|  | 14 | <LoQ | <LoQ | <LoQ | <LoQ | |  |
|  | 0 | 50,4 | 49,6 | 0,8 | 0,0 | | 100 |
|  | 1 | 51,7 | 48,3 | 3,5 | -2,7 | | 37 |
|  | 4 | 45,5 | 54,5 | -9,1 | 9,9 | | 3 |
|  | 7 | 52,7 | 47,3 | 5,4 | -4,7 | | 2 |
|  | 14 | <LoQ | <LoQ | <LoQ | <LoQ | |  |
| 13 | 0 | 50,2 | 49,8 | 0,4 | 0,1 | | 70 |
|  | 1 | <LoQ | <LoQ | <LoQ | <LoQ | |  |
|  | 3 | <LoQ | <LoQ | <LoQ | <LoQ | |  |
|  | 7 | <LoQ | <LoQ | <LoQ | <LoQ | |  |
|  | 14 | <LoQ | <LoQ | <LoQ | <LoQ | |  |
|  | 0 | 50,2 | 49,8 | 0,5 | 0,0 | | 100 |
|  | 1 | 49,6 | 50,4 | -0,9 | 1,4 | | 13 |
|  | 3 | <LoQ | <LoQ | <LoQ | <LoQ | |  |
|  | 7 | <LoQ | <LoQ | <LoQ | <LoQ |  | |
|  | 14 | <LoQ | <LoQ | <LoQ | <LoQ |  | |
| 14 | 0 | 48,1 | 51,9 | -3,9 | 0,0 | 20 | |
|  | 1 | <LoQ | <LoQ | <LoQ | <LoQ | <LoQ | |
|  | 3 | <LoQ | <LoQ | <LoQ | <LoQ | <LoQ | |
|  | 7 | <LoQ | <LoQ | <LoQ | <LoQ | <LoQ | |
|  | 14 | <LoQ | <LoQ | <LoQ | <LoQ | <LoQ | |
|  | 0 | 49,6 | 50,4 | -0,8 | -3,1 | 100 | |
|  | 1 | 51,6 | 48,4 | 3,3 | -7,2 | 4 | |
|  | 3 | <LoQ | <LoQ | <LoQ | <LoQ | <LoQ | |
|  | 7 | <LoQ | <LoQ | <LoQ | <LoQ | <LoQ | |
|  | 14 | <LoQ | <LoQ | <LoQ | <LoQ | <LoQ | |
| 15 | 0 | 49,5 | 50,5 | -1,1 | 1,4 | 81 | |
|  | 1 | <LoQ | <LoQ | <LoQ | <LoQ | <LoQ | |
|  | 3 | <LoQ | <LoQ | <LoQ | <LoQ | <LoQ | |
|  | 7 | <LoQ | <LoQ | <LoQ | <LoQ | <LoQ | |
|  | 14 | <LoQ | <LoQ | <LoQ | <LoQ | <LoQ | |
|  | 0 | 50,2 | 49,8 | 0,3 | 0,0 | 100 | |
|  | 1 | <LoQ | <LoQ | <LoQ | <LoQ | <LoQ | |
|  | 3 | <LoQ | <LoQ | <LoQ | <LoQ | <LoQ | |
|  | 7 | <LoQ | <LoQ | <LoQ | <LoQ | <LoQ | |
|  | 14 | <LoQ | <LoQ | <LoQ | <LoQ | <LoQ | |
| 16 | 0 | 50,0 | 50,0 | 0,0 | 0,0 | 100 | |
|  | 1 | 49,5 | 50,5 | -1,1 | 1,1 | 56 | |
|  | 2 | 51,2 | 48,8 | 2,5 | -2,5 | 11 | |
|  | 7 | <LoQ | <LoQ | <LoQ | <LoQ | <LoQ | |
| 17 | 0 | 50,0 | 50,0 | -0,1 | -0,9 | 82 | |
|  | 1 | 51,0 | 49,0 | 2,1 | -3,1 | 17 | |
|  | 3 | <LoQ | <LoQ | <LoQ | <LoQ | <LoQ | |
|  | 7 | <LoQ | <LoQ | <LoQ | <LoQ | <LoQ | |
|  | 14 | <LoQ | <LoQ | <LoQ | <LoQ | <LoQ | |
|  | 0 | 49,5 | 50,5 | -1,0 | 0,0 | 100 | |
|  | 1 | 53,2 | 46,8 | 6,4 | -7,4 | 18 | |
|  | 3 | <LoQ | <LoQ | <LoQ | <LoQ | <LoQ | |
|  | 7 | <LoQ | <LoQ | <LoQ | <LoQ | <LoQ | |
|  | 13 | <LoQ | <LoQ | <LoQ | <LoQ | <LoQ | |
| 18 | 0 | 51,4 | 48,6 | 2,7 | 0,0 | 100 | |
|  | 1 | 52,2 | 47,8 | 4,5 | -1,8 | 30 | |
|  | 2 | 50,5 | 49,5 | 0,9 | 1,8 | 19 | |
|  | 7 | <LoQ | <LoQ | <LoQ | <LoQ | <LoQ | |
|  | 14 | <LoQ | <LoQ | <LoQ | <LoQ | <LoQ | |
|  | 0 | 50,8 | 49,2 | 1,7 | 1,0 | 54 | |
|  | 1 | <LoQ | <LoQ | <LoQ | <LoQ | <LoQ | |
|  | 2 | <LoQ | <LoQ | <LoQ | <LoQ | <LoQ | |
|  | 7 | <LoQ | <LoQ | <LoQ | <LoQ | <LoQ | |
|  | 14 | <LoQ | <LoQ | <LoQ | <LoQ | <LoQ | |

**Compound 4**

| Study | DAF | Molar fraction Isomer 1  [%] | Molar fraction Isomer 2  [%] | Stereoisomeric excess (SE) [%] | SE change [%] | | Amount expressed as percentage of highest residue measured in study [%] |
| --- | --- | --- | --- | --- | --- | --- | --- |
| 1 | 0 | 50,2 | 49,8 | 0,5 | 0 | | 100 |
|  | 1 | 50,2 | 49,8 | 0,3 | 0,2 | | 77 |
|  | 3 | 48,3 | 51,7 | -3,4 | 3,9 | | 17 |
|  | 7 | 46,0 | 54,0 | -8,1 | 8,6 | | 9 |
|  | 10 | <LoQ | <LoQ | <LoQ | <LoQ | | 0 |
| 2 | 0 | 50,0 | 50,0 | 0,0 | 0 | | 100 |
|  | 1 | 50,3 | 49,7 | 0,6 | -0,6 | | 20 |
|  | 3 | <LoQ | <LoQ | <LoQ | <LoQ | | 0 |
|  | 7 | <LoQ | <LoQ | <LoQ | <LoQ | | 0 |
|  | 14/-0 | <LoQ | <LoQ | <LoQ | <LoQ | | 0 |
|  | 0 | 50,1 | 49,9 | 0,2 | -0,2 | | 66 |
|  | 1 | 50,1 | 49,9 | 0,2 | -0,2 | | 35 |
|  | 4 | <LoQ | <LoQ | <LoQ | <LoQ | | 0 |
|  | 7 | <LoQ | <LoQ | <LoQ | <LoQ | | 0 |
|  | 14 | <LoQ | <LoQ | <LoQ | <LoQ | | 0 |
| 3 | 0 | 49,5 | 50,5 | -1,0 | -2,0 | | 62 |
|  | 1 | 49,3 | 50,7 | -1,5 | -1,6 | | 15 |
|  | 3 | <LoQ | <LoQ | <LoQ | <LoQ | | 0 |
|  | 7 | <LoQ | <LoQ | <LoQ | <LoQ | | 0 |
|  | 14/-0 | <LoQ | <LoQ | <LoQ | <LoQ | | 0 |
|  | 0 | 48,5 | 51,5 | -3,0 | 0,0 | | 100 |
|  | 1 | 48,9 | 51,1 | -2,2 | -0,8 | | 79 |
|  | 3 | 46,4 | 53,6 | -7,1 | 4,1 | | 14 |
|  | 7 | <LoQ | <LoQ | <LoQ | <LoQ | | 0 |
|  | 14 | <LoQ | <LoQ | <LoQ | <LoQ | | 0 |
| 4 | 0 | 50,1 | 49,9 | 0,2 | -0,2 | | 71 |
|  | 1 | 48,2 | 51,8 | -3,6 | 3,6 | | 16 |
|  | 14/-0 | <LoQ | <LoQ | <LoQ | <LoQ | | 0 |
|  | 0 | 50,0 | 50,0 | 0,0 | 0,0 | | 100 |
|  | 1 | 49,1 | 50,9 | -1,7 | 1,7 | 27 | |
|  | 3 | 47,9 | 52,1 | -4,1 | 4,1 | 9 | |
|  | 7 | <LoQ | <LoQ | <LoQ | <LoQ | 0 | |
|  | 10 | <LoQ | <LoQ | <LoQ | <LoQ | 0 | |
| 5 | 0 | 50,3 | 49,7 | 0,7 | 0 | 100 | |
|  | 1 | 50,3 | 49,7 | 0,5 | 0,2 | 18 | |
|  | 2 | 49,8 | 50,2 | -0,4 | 1,0 | 14 | |
|  | 8 | <LoQ | <LoQ | <LoQ | <LoQ | 0 | |
|  | 9 | <LoQ | <LoQ | <LoQ | <LoQ | 0 | |
| 6 | 0 | 50,3 | 49,7 | 0,7 | 0,0 | 46 | |
|  | 21/-0 | <LoQ | <LoQ | <LoQ | <LoQ | 0 | |
|  | 0 | 50,3 | 49,7 | 0,6 | 0,0 | 100 | |
|  | 1 | 50,2 | 49,8 | 0,5 | 0,1 | 32 | |
|  | 3 | 48,2 | 51,8 | -3,7 | 4,3 | 14 | |
|  | 7 | <LoQ | <LoQ | <LoQ | <LoQ | 0 | |
| 7 | 0 | 49,6 | 50,4 | -0,8 | 1,6 | 74 | |
|  | 1 | 49,9 | 50,1 | -0,3 | 1,1 | 34 | |
|  | 3 | 49,5 | 50,5 | -1,0 | 1,8 | 13 | |
|  | 10/-0 | <LoQ | <LoQ | <LoQ | <LoQ | 0 | |
|  | 0 | 50,4 | 49,6 | 0,8 | 0,0 | 100 | |
|  | 1 | 50,5 | 49,5 | 1,0 | -0,2 | 33 | |
|  | 3 | <LoQ | <LoQ | <LoQ | <LoQ | 0 | |
|  | 10/-0 | <LoQ | <LoQ | <LoQ | <LoQ | 0 | |
|  | 0 | 50,2 | 49,8 | 0,3 | 0,5 | 59 | |
|  | 1 | 50,5 | 49,5 | 1,1 | -0,3 | 33 | |
|  | 3 | 51,3 | 48,7 | 2,5 | -1,7 | 11 | |
|  | 5 | 49,3 | 50,7 | -1,4 | 2,2 | 10 | |
|  | 7 | 50,8 | 49,2 | 1,6 | -0,8 | 10 | |
|  | 9 | 51,3 | 48,7 | 2,6 | -1,8 | 9 | |
| 8 | 0 | 51,0 | 49,0 | 1,9 | -0,2 | 84 | |
|  | 1 | 50,7 | 49,3 | 1,3 | 0,4 | 74 | |
|  | 16/-0 | <LoQ | <LoQ | <LoQ | <LoQ | 0 | |
|  | 0 | 50,9 | 49,1 | 1,7 | 0,0 | 100 | |
|  | 1 | 50,9 | 49,1 | 1,7 | 0,0 | 43 | |
|  | 4 | 46,6 | 53,4 | -6,7 | 8,4 | 21 | |
|  | 7 | 47,3 | 52,7 | -5,4 | 7,1 | 15 | |
|  | 11 | 44,6 | 55,4 | -10,8 | 12,6* | 7* | |
| 9 | 0 | 50,2 | 49,8 | 0,4 | 0,3 | 76 | |
|  | 1 | <LoQ | <LoQ | <LoQ | <LoQ |  | |
|  | 3 | <LoQ | <LoQ | <LoQ | <LoQ |  | |
|  | 10/-0 | <LoQ | <LoQ | <LoQ | <LoQ |  | |
|  | 0 | 50,2 | 49,8 | 0,5 | 0,2 | 82 | |
|  | 1 | <LoQ | <LoQ | <LoQ | <LoQ |  | |
|  | 3 | <LoQ | <LoQ | <LoQ | <LoQ |  | |
|  | 7 | <LoQ | <LoQ | <LoQ | <LoQ |  | |
|  | 10/-0 | <LoQ | <LoQ | <LoQ | <LoQ |  | |
|  | 0 | 50,3 | 49,7 | 0,7 | 0,0 | 100 | |
|  | 1 | 51,2 | 48,8 | 2,4 | -1,7 | 25 | |
|  | 3 | 53,0 | 47,0 | 6,0 | -5,3 | 8 | |
|  | 5 | <LoQ | <LoQ | <LoQ | <LoQ |  | |
|  | 7 | <LoQ | <LoQ | <LoQ | <LoQ | |  |
|  | 10 | <LoQ | <LoQ | <LoQ | <LoQ | |  |
| 10 | 0 | <LoQ | <LoQ | <LoQ | <LoQ | |  |
|  | 1 | 49,8 | 50,2 | -0,4 | 0,4 | | 76 |
|  | 14 | <LoQ | <LoQ | <LoQ | <LoQ | |  |
|  | 0 | 50,0 | 50,0 | 0,0 | 0,0 | | 100 |
|  | 1 | 50,0 | 50,0 | 0,0 | 0,0 | | 99 |
|  | 3 | 50,0 | 50,0 | -0,1 | 0,1 | | 23 |
|  | 7 | <LoQ | <LoQ | <LoQ | <LoQ | |  |
|  | 9 | <LoQ | <LoQ | <LoQ | <LoQ | |  |
| 11 | 0 | 49,9 | 50,1 | -0,2 | 0 | | 100 |
|  | 1 | 49,7 | 50,3 | -0,7 | 0,4 | | 66 |
|  | 3 | 50,0 | 50,0 | -0,1 | -0,1 | | 42 |
|  | 7/-0 | 50,4 | 49,6 | 0,8 | -1,1 | | 66 |
|  | 1 | 50,9 | 49,1 | 1,9 | -2,1 | | 14 |
| 12 | 0 | 49,4 | 50,6 | -1,3 | 0,9 | | 69 |
|  | 10 | 49,8 | 50,2 | -0,4 | 0,0 | | 100 |
|  | 0 | 49,1 | 50,9 | -1,8 | -1,4 | | 64 |
|  | 1 | 49,1 | 50,9 | -1,7 | 1,3 | | 22 |
|  | 3 | 48,7 | 51,3 | -2,7 | 2,3 | | 31 |
| 13 | 0 | 51,0 | 49,0 | 2,0 | 0 | | 100 |
|  | 1 | 51,9 | 48,1 | 3,7 | -1,7 | | 53 |
|  | 3 | 55,1 | 44,9 | 10,1 | -8,1 | | 30 |
|  | 7 | 60,8 | 39,2 | 21,7 | -19,7 | | 18 |
|  | 10 | 60,8 | 39,2 | 21,6 | -19,6 | | 16 |
|  | 14/-0 | 60,9 | 39,1 | 21,8 | -19,8* | | 6* |
|  | 0 | 52,2 | 47,8 | 4,3 | -2,3 | | 82 |
|  | 1 | 52,8 | 47,2 | 5,7 | -3,7 | | 48 |
|  | 3 | 53,5 | 46,5 | 7,1 | -5,1 | | 32 |
|  | 7 | 54,2 | 45,8 | 8,4 | -6,4 | 16 | |
|  | 10 | 55,2 | 44,8 | 10,4 | -8,4 | 8 | |
| 14 | 0 | 50,6 | 49,4 | 1,1 | 0,3 | 92 | |
|  | 1 | 50,5 | 49,5 | 1,1 | 0,3 | 54 | |
|  | 3 | <LoQ | <LoQ | <LoQ | <LoQ | <LoQ | |
|  | 7 | <LoQ | <LoQ | <LoQ | <LoQ | <LoQ | |
|  | 14/-0 | <LoQ | <LoQ | <LoQ | <LoQ | <LoQ | |
|  | 0 | 50,7 | 49,3 | 1,4 | 0,0 | 100 | |
|  | 1 | 50,9 | 49,1 | 1,7 | -0,3 | 42 | |
|  | 3 | <LoQ | <LoQ | <LoQ | <LoQ | <LoQ | |
|  | 7 | 58,6 | 41,4 | 17,2 | -15,9 | 21 | |
|  | 14 | <LoQ | <LoQ | <LoQ | <LoQ | <LoQ | |
| 15 | 0 | 49,6 | 50,4 | -0,9 | 9,3 | 43 | |
|  | 1 | 51,1 | 48,9 | 2,1 | 6,3 | 4 | |
|  | 3 | <LoQ | <LoQ | <LoQ | <LoQ | <LoQ | |
|  | 7 | <LoQ | <LoQ | <LoQ | <LoQ | <LoQ | |
|  | 14 | <LoQ | <LoQ | <LoQ | <LoQ | <LoQ | |
|  | 0 | 50,4 | 49,6 | 0,9 | 7,5 | 100 | |
|  | 1 | 51,1 | 48,9 | 2,3 | 6,1 | 19 | |
|  | 3 | 51,3 | 48,7 | 2,5 | 5,9 | 11 | |
|  | 7 | 52,3 | 47,7 | 4,6 | 3,8 | 6 | |
|  | 14 | <LoQ | <LoQ | <LoQ | <LoQ | <LoQ | |
| 16 | 0 | 49,4 | 50,6 | -1,2 | 0,0 | 100 | |
|  | 1 | 49,6 | 50,4 | -0,8 | -0,3 | 70 | |
|  | 2 | 50,4 | 49,6 | 0,8 | -2,0 | 49 | |
|  | 7 | 52,3 | 47,7 | 4,7 | -5,9 | 8 | |
| 17 | 0 | 49,4 | 50,6 | -1,1 | 0,0 | 100 | |
|  | 1 | 48,9 | 51,1 | -2,3 | 1,1 | 34 | |
|  | 3 | 47,0 | 53,0 | -5,9 | 4,8 | 10 | |
|  | 7 | <LoQ | <LoQ | <LoQ | <LoQ | <LoQ | |
|  | 14 | <LoQ | <LoQ | <LoQ | <LoQ | <LoQ | |
|  | 0 | 49,0 | 51,0 | -1,9 | 0,8 | 40 | |
|  | 1 | 48,3 | 51,7 | -3,4 | 2,2 | 22 | |
|  | 3 | 46,5 | 53,5 | -6,9 | 5,8 | 15 | |
|  | 7 | <LoQ | <LoQ | <LoQ | <LoQ | <LoQ | |
|  | 13 | <LoQ | <LoQ | <LoQ | <LoQ | <LoQ | |
| 18 | 0 | 50,3 | 49,7 | 0,7 | 0,0 | 100 | |
|  | 1 | 48,4 | 51,6 | -3,2 | 3,8 | 35 | |
|  | 2 | 48,5 | 51,5 | -3,0 | 3,6 | 28 | |
|  | 7 | <LoQ | <LoQ | <LoQ | <LoQ | <LoQ | |
|  | 14 | <LoQ | <LoQ | <LoQ | <LoQ | <LoQ | |
|  | 0 | 50,6 | 49,4 | 1,3 | -0,6 | 65 | |
|  | 1 | 48,1 | 51,9 | -3,9 | 4,5 | 16 | |
|  | 2 | <LoQ | <LoQ | <LoQ | <LoQ | <LoQ | |
|  | 7 | <LoQ | <LoQ | <LoQ | <LoQ | <LoQ | |
|  | 14 | <LoQ | <LoQ | <LoQ | <LoQ | <LoQ | |

**Compound 5**

| Study | DAF | Molar fraction Diastereomer 1  [%] | Molar fraction Diastereomer 2  [%] | Stereoisomeric excess (SE) [%] | SE change [%] | Amount expressed as percentage of highest residue measured in study [%] |
| --- | --- | --- | --- | --- | --- | --- |
| 1*** | 0 | 56,6 | 43,4 | 13,2 | -2,6 | 83 |
|  | 1 | 57,5 | 42,5 | 15,0 | -4,4 | 53 |
|  | 3 | 58,8 | 41,2 | 17,7 | -7,1 | 20 |
|  | 6 | 59,8 | 40,2 | 19,7 | -9,1 | 15 |
|  | 10 | 59,2 | 40,8 | 18,3 | -7,8 | 12 |
|  | 0 | 55,3 | 44,7 | 10,6 | 0,0 | 100 |
|  | 1 | 56,6 | 43,4 | 13,2 | -2,6 | 54 |
|  | 10 | 60,3 | 39,7 | 20,5 | -10,0 | 12 |
|  | 0 | 57,6 | 42,4 | 15,2 | -4,6 | 75 |
|  | 1 | 57,7 | 42,3 | 15,4 | -4,8 | 39 |
|  | 3 | 57,9 | 42,1 | 15,9 | -5,3 | 26 |
|  | 7 | 61,1 | 38,9 | 22,2 | -11,7 | 18 |
|  | 10 | 62,9 | 37,1 | 25,8 | -15,3* | 8* |
|  | 15 | 66,9 | 33,1 | 33,8 | -23,2* | 3* |
|  | 25 | 0.0031 | <0.0025 | <LoQ | <LoQ | <LoQ |
|  | 35 | <0.0025 | <0.0025 | <LoQ | <LoQ | <LoQ |
| 2*** | 0 | 58,8 | 41,2 | 17,7 | -7,2 | 55 |
|  | 1 | 57,9 | 42,1 | 15,9 | -5,4 | 8 |
|  | 3 | 60,8 | 39,2 | 21,6 | -11,2* | 3* |
|  | 6 | 58,0 | 42,0 | 16,1 | -5,6 | 2 |
|  | 10 | <LoQ | <LoQ | <LoQ | <LoQ | <LoQ |
|  | 0 | 55,2 | 44,8 | 10,4 | 0,0 | 100 |
|  | 1 | 57,9 | 42,1 | 15,7 | -5,3 | 15 |
|  | 10 | <LoQ | <LoQ | <LoQ | <LoQ | <LoQ |
|  | 0 | 56,5 | 43,5 | 13,0 | -2,6 | 91 |
|  | 1 | 60,1 | 39,9 | 20,2 | -9,8 | 18 |
|  | 3 | 61,1 | 38,9 | 22,2 | -11,8* | 7* |
|  | 7 | 61,9 | 38,1 | 23,7 | -13,3* | 4* |
|  | 10 | 64,1 | 35,9 | 28,1 | -17,7* | 3* |
|  | 15 | <LoQ | <LoQ | <LoQ | <LoQ | <LoQ |
|  | 25 | <LoQ | <LoQ | <LoQ | <LoQ | <LoQ |
|  | 35 | <LoQ | <LoQ | <LoQ | <LoQ | <LoQ |
| 3*** | 0 | 65,5 | 34,5 | 31,1 | 0 | 100 |
|  | 1 | 63,5 | 36,5 | 27,0 | 4,0 | 45 |
|  | 3 | 65,9 | 34,1 | 31,8 | -0,7 | 25 |
|  | 7 | 64,2 | 35,8 | 28,3 | 2,7 | 7,7 |
|  | 14 | <LoQ | <LoQ | <LoQ | <LoQ | <LoQ |
|  | 0 | 65,0 | 35,0 | 30,0 | 1,1 | 99 |
|  | 1 | 61,6 | 38,4 | 23,1 | 7,9 | 18 |
|  | 3 | 61,2 | 38,8 | 22,3 | 8,8 | 12 |
|  | 7 | 57,4 | 42,6 | 14,8 | 16,3* | 3* |
|  | 14 | <LoQ | <LoQ | <LoQ | <LoQ | <LoQ |
|  | 21 | <LoQ | <LoQ | <LoQ | <LoQ | <LoQ |
|  | 28 | <LoQ | <LoQ | <LoQ | <LoQ | <LoQ |
| 4*** | 0 | 61,1 | 38,9 | 22,2 | -8,3 | 46 |
|  | 1 | 54,2 | 45,8 | 8,4 | 5,5 | 8 |
|  | 4 | <LoQ | <LoQ | <LoQ | <LoQ | <LoQ |
|  | 8 | <LoQ | <LoQ | <LoQ | <LoQ | <LoQ |
|  | 14 | <LoQ | <LoQ | <LoQ | <LoQ | <LoQ |
|  | 0 | 57,0 | 43,0 | 13,9 | 0,0 | 100 |
|  | 1 | 60,4 | 39,6 | 20,7 | -6,8 | 39 |
|  | 3 | 61,1 | 38,9 | 22,3 | -8,4 | 20 |
|  | 7 | 61,4 | 38,6 | 22,7 | -8,8 | 10 |
|  | 14 | 59,0 | 41,0 | 18,0 | -4,1 | 7 |
|  | 21 | 60,0 | 40,0 | 20,1 | -6,2 | 5 |
|  | 28 | <LoQ | <LoQ | <LoQ | <LoQ | <LoQ |
| 5 | 0 | 56,1 | 43,9 | 12,2 | 0,9 | 100 |
|  | 1 | 58,0 | 42,0 | 16,1 | -2,9 | 36 |
|  | 3 | 59,9 | 40,1 | 19,8 | -6,6 | 20 |
|  | 7 | 61,5 | 38,5 | 22,9 | -9,7 | 12 |
|  | 9 | 60,6 | 39,4 | 21,2 | -8,0 | 11 |
|  | 0 | 56,6 | 43,4 | 13,2 | 0,0 | 100 |
|  | 1 | 58,5 | 41,5 | 16,9 | -3,7 | 29 |
|  | 3 | 59,9 | 40,1 | 19,7 | -6,5 | 21 |
|  | 7 | 61,5 | 38,5 | 22,9 | -9,7 | 12 |
|  | 10 | 59,3 | 40,7 | 18,5 | -5,3 | 10 |
|  | 14 | 60,8 | 39,2 | 21,5 | -8,3 | 7 |
|  | 21 | 65,2 | 34,8 | 30,3 | -17,1* | 4* |
|  | 28 | 66,3 | 33,7 | 32,6 | -19,4* | 2* |
| 6 | 0 | 60,8 | 39,2 | 21,6 | -1,5 | 66 |
|  | 1 | 62,5 | 37,5 | 24,9 | -4,8 | 19 |
|  | 14 | <LoQ | <LoQ | <LoQ | <LoQ | 0 |
|  | 0 | 60,1 | 39,9 | 20,1 | 0,0 | 100 |
|  | 1 | 61,9 | 38,1 | 23,9 | -3,8 | 35 |
|  | 3 | 61,7 | 38,3 | 23,4 | -3,3 | 14 |
|  | 7 | 62,3 | 37,7 | 24,5 | -4,4 | 5 |
|  | 10 | <LoQ | <LoQ | <LoQ | <LoQ | 0 |
| 7 | 0 | 57,6 | 42,4 | 15,3 | 0 | 100 |
|  | 1 | 59,3 | 40,7 | 18,7 | -3,4 | 9 |
|  | 2 | 61,5 | 38,5 | 23,0 | -7,7 | 7 |
|  | 8 | <LoQ | <LoQ | <LoQ | <LoQ | 0 |
|  | 9 | <LoQ | <LoQ | <LoQ | <LoQ | 0 |
| 8 | 0 | 58,9 | 41,1 | 17,7 | 0 | 100 |
|  | 1 | 58,8 | 41,2 | 17,6 | 0,1 | 64 |
|  | 3 | 58,2 | 41,8 | 16,5 | 1,3 | 28 |
|  | 7 | <LoQ | <LoQ | <LoQ | <LoQ | 7 |
|  | 10 | <LoQ | <LoQ | <LoQ | <LoQ | 0 |
| 9 | 0 | 61,1 | 38,9 | 22,3 | 0,0 | 100 |
|  | -0 | 62,3 | 37,7 | 24,5 | -2,3 | 4 |
|  | 0 | 60,8 | 39,2 | 21,6 | 0,7 | 88 |
|  | 1 | 65,2 | 34,8 | 30,4 | -8,1 | 17 |
|  | 3 | 65,1 | 34,9 | 30,1 | -7,9 | 12 |
|  | 5 | 57,6 | 42,4 | 15,1 | 7,2 | 3 |
|  | 7 | 62,6 | 37,4 | 25,1 | -2,8 | 5 |
|  | 14 | 58,1 | 41,9 | 16,3 | 6,0 | 3 |
|  | 21 | 60,0 | 40,0 | 19,9 | 2,4 | 5 |
|  | 28 | 59,1 | 40,9 | 18,2 | 4,1 | 7 |
|  | 35 | 60,2 | 39,8 | 20,4 | 1,9 | 10 |
| 10 | 0 | 58,7 | 41,3 | 17,4 | 0,0 | 100 |
|  | -0 | 63,9 | 36,1 | 27,8 | -10,4 | 21 |
|  | 0 | 57,5 | 42,5 | 15,1 | 2,4 | 98 |
|  | 1 | 60,9 | 39,1 | 21,8 | -4,4 | 29 |
|  | 3 | 62,7 | 37,3 | 25,4 | -8,0 | 12 |
|  | 5 | 63,5 | 36,5 | 27,1 | -9,6 | 15 |
|  | 7 | 63,9 | 36,1 | 27,9 | -10,4 | 14 |
|  | 14 | 58,6 | 41,4 | 17,2 | 0,2 | 11 |
|  | 21 | 59,5 | 40,5 | 19,1 | -1,6 | 4 |
|  | 28 | 61,4 | 38,6 | 22,9 | -5,4 | 13 |
|  | 35 | 58,4 | 41,6 | 16,9 | 0,6 | 11 |
| 11 | 0 | 58,6 | 41,4 | 17,2 | -3,9 | 58 |
|  | -0 | 60,9 | 39,1 | 21,8 | -8,6 | 0 |
|  | 0 | 56,6 | 43,4 | 13,3 | 0,0 | 100 |
|  | 1 | 58,8 | 41,2 | 17,6 | -4,3 | 17 |
|  | 3 | 64,0 | 36,0 | 28,0 | -14,7* | 1* |
|  | 5 | 63,0 | 37,0 | 26,1 | -12,8* | 1* |
|  | 7 | 62,7 | 37,3 | 25,4 | -12,1* | 1* |
|  | 14 | 62,1 | 37,9 | 24,3 | -11,0* | 0* |
|  | 21 | 59,9 | 40,1 | 19,8 | -6,5 | 0 |
|  | 28 | 61,5 | 38,5 | 23,1 | -9,8 | 0 |
|  | 35 | 61,1 | 38,9 | 22,2 | -8,9 | 0 |

*** in these studies, only the diastereomers were measured, not all single isomers
